# M2a macrophages facilitate resolution of chemically-induced colitis in TLR4-SNP mice

**DOI:** 10.1128/mbio.01208-23

**Published:** 2023-09-28

**Authors:** Alexandra M. Vlk, Daniel Prantner, Kari Ann Shirey, Darren J. Perkins, Marguerite S. Buzza, Vivek Thumbigere-Math, Achsah D. Keegan, Stefanie N. Vogel

**Affiliations:** 1 Department of Microbiology and Immunology, University of Maryland School of Medicine, Baltimore, Maryland, USA; 2 University of Maryland Marlene & Stewart Greenebaum Comprehensive Cancer Center, Baltimore, Maryland, USA; 3 Department of Physiology, University of Maryland School of Medicine, Baltimore, Maryland, USA; 4 Center for Vascular and Inflammatory Diseases, University of Maryland School of Medicine, Baltimore, Maryland, USA; 5 Division of Periodontics, University of Maryland School of Dentistry, Baltimore, Maryland, USA; School of Medicine, Oregon Health & Science University, Portland, Oregon, USA

**Keywords:** TLR4 SNPs, DSS-induced colitis, M2a macrophages, IL-4Rα, PPARγ, rosiglitazone

## Abstract

Toll-like receptor 4 (TLR4) is an innate immune receptor responsive to lipopolysaccharide (LPS). Single nucleotide polymorphisms (SNPs) in human TLR4 that encode an A896G transition at SNP rs4986790 (D299G) and a C1196T transition at SNP rs4986791 (T399I) render individuals hyporesponsive to LPS. In humans, these SNPs are also associated with increased susceptibility to inflammatory bowel diseases (IBDs). Using knock-in mice engineered to express the murine homologs of these human TLR4 mutations (“TLR4-SNP” mice), we have shown that TLR4-SNP mice develop significantly more severe colitis induced by dextran sodium sulfate (DSS) than wild-type (WT) mice, similar to IBD in humans expressing these SNPs. Previous studies have provided indirect evidence for “tissue repair” M2 macrophages (Mφ) in the resolution of colitis. Signaling through the IL-4/IL-13 receptor, IL-4Rα, and the transcription factor, peroxisome proliferator-activated receptor (PPARγ), have been shown to be required for induction of M2a Mφ, and our data provide direct evidence for the involvement of both in the repair of DSS-induced colonic damage. In response to DSS, colons of TLR4-SNP mice produced reduced levels of M2a Mφ marker mRNA and protein, including PPARγ, and therapeutic administration of the PPARγ agonist ligand, rosiglitazone, resolved colitis in TLR4-SNP mice, and increased expression of the M2a protein, Ym1. Together, these data indicate that the failure of TLR4-SNP mice to resolve DSS-induced colitis may be secondary to their failure to induce “tissue repair” M2a Mφ.

**Importance:**

Inflammatory bowel disease (IBD), including Crohn’s disease and ulcerative colitis, impacts millions of individuals worldwide and severely impairs the quality of life for patients. Dysregulation of innate immune signaling pathways reduces barrier function and exacerbates disease progression. Macrophage (Mφ) signaling pathways are potential targets for IBD therapies. While multiple treatments are available for IBD, (i) not all patients respond, (ii) responses may diminish over time, and (iii) treatments often have undesirable side effects. Genetic studies have shown that the inheritance of two co-segregating SNPs expressed in the innate immune receptor, TLR4, is associated with human IBD. Mice expressing homologous SNPs (“TLR4-SNP” mice) exhibited more severe colitis than WT mice in a DSS-induced colonic inflammation/repair model. We identified a critical role for M2a “tissue repair” Mφ in the resolution of colitis. Our findings provide insight into potential development of novel therapies targeting Mφ signaling pathways that aim to alleviate the debilitating symptoms experienced by individuals with IBD.

## INTRODUCTION

Toll-like receptor 4 (TLR4) is a pattern recognition receptor (PRR) predominantly expressed on innate immune cells, such as macrophages (Mφ) and dendritic cells, and responds to the prototype ligand, Gram-negative lipopolysaccharide (LPS) (1, 2), as well as to other microbial “pathogen-associated molecular patterns” (PAMPs) and host-derived “danger-associated molecular patterns” (DAMPs) (3 – 5). TLR4 is unique in that (i) it requires a co-receptor, MD-2, for ligand binding and dimerization, and (ii) in response to ligand, TLR4 activates two distinct intracellular signaling pathways, the MyD88 and TRIF signaling pathways (6, 7). Activation of these two pathways results in transcriptional responses leading to production of cytokines, chemokines, and interferons. While TLR4-mediated inflammatory responses to infection or trauma are intended to protect the host, an overexuberant response may lead to tissue damage.

Single nucleotide polymorphisms (SNPs) in human *TLR4*, an A896G transition at SNP rs4986790 (D299G) and a C1196T transition at SNP rs4986791 (T399I), were originally reported to cause TLR4 hyporesponsiveness to LPS in humans (8). Human TLR4 proteins containing the D299G and the co-segregating D299G/T399I mutations in the extracellular domain have since been associated with increased susceptibility to many diseases, e.g., Gram negative sepsis (9), inflammatory bowel disease (IBD) (10), respiratory syncytial virus (RSV) (11 – 14), and others (15 – 21). In other disease models, expression of these same SNPs is known to be protective, including in influenza virus infection (14), graft-versus-host disease (22), atherogenesis (23), acute coronary syndrome (24), rheumatoid arthritis (25), and leprosy (26).

Expression of the D299G/T399I SNPs in TLR4 affects its functionality (8, 14, 27), possibly by altering TLR4 expression and/or by interfering with TLR4 dimerization and recruitment of adapter molecules (8, 14, 27 – 32). The D299G mutation is thought to have arisen in Africa because it offered protection from malaria, while the T399I mutation likely emerged in individuals who migrated from Africa to Europe under selective pressure exerted by the plague (33). Genetic analyses showed that the D299G/T399I SNPs, expressed in the ectodomain of human TLR4, often co-segregate with a carrier frequency of 10%–18% in Caucasians (33). Our laboratory engineered a knock-in mouse strain that homozygously expresses homologous SNPs (D298G/N397I) in murine TLR4, and this model displays similar LPS-hyporesponsiveness and altered susceptibility to infection as previously reported in human studies (14).

IBD, comprising Crohn’s disease (CD) and ulcerative colitis (UC), impacts millions globally (34) and presents with relapsing and remitting colonic inflammation that interferes with quality of life (35, 36). Sex differences exist in IBD prevalence, with early onset CD (<16 years) being slightly more common in males, and UC occurring more frequently in older males (>45 years) (37). Controlling inflammation is challenging and although a variety of treatments are available (38 – 40), not all patients respond to treatment and treatment efficacy can wane over time, highlighting the need for new, and possibly individualized, therapeutics to improve IBD management and patient outcomes.

Even prior to the identification of TLR4 as the LPS sensor (1, 2), inbred mouse strains that exhibited LPS unresponsiveness (e.g., C3H/HeJ and C57BL/10ScN) (41, 42) also differed in susceptibility to dextran sodium sulfate (DSS)-induced colitis (43, 44). It was later discovered that these two mouse strains had naturally occurring mutations in the *Tlr4* gene (1, 2). Subsequently, LPS-unresponsive TLR4^−/−^ mice (45) were shown to develop more severe DSS-induced colitis than WT mice (46, 47). TLR4 has been reported to be upregulated in Mφ and intestinal epithelial cells during intestinal inflammation (48 – 50). Although the connection between impaired TLR4 sensing and increased sensitivity to IBD was noted many years ago, the underlying mechanisms remain elusive.

Main contributors to the complexity of IBD pathogenesis include dysregulation of the mucosal immune system, alteration of the gut microflora, and impairment of the epithelial barrier function (38, 39, 51 – 53). IL-10-deficient mice develop spontaneous colitis associated with an exuberant Th1 response and uncontrolled cytokine production by Mφ (54). The inflammatory response associated with IBD has been attributed to the action of Th17 cells (38, 39, 55, 56). Functioning cellular programs for intestinal homeostasis, immune tolerance, and epithelial repair are key to maintaining gut health (53). Genome-wide association studies have identified IBD susceptibility loci that are related to epithelial barrier function and innate and adaptive immunity (38, 53, 57), emphasizing the importance of genetic predisposition and the likely polygenic control of IBD, including genetic variation in TLRs (58, 59). However, the precise mechanism(s) by which the TLR4 299/399 SNPs increase sensitivity to IBD in humans is unknown.

Herein, we show that, like humans with IBD, the TLR4-SNP mice exhibit much more severe colitis in response to DSS than WT mice. Using IL-4Rα^−/−^ mice and mice with a conditional deletion of PPARγ in myeloid cells (PPARγ^cKO^), that fail to respond normally to IL-4 or IL-13 to induce Mφ with an M2a “repair” phenotype, we show that both IL-4Rα- and PPARγ-dependent signaling are required for amelioration of DSS-induced colonic tissue injury. DSS-treated TLR4-SNP mice exhibited a reduced capacity to express M2a Mφ markers, and their colitic disease was ameliorated by therapeutic treatment with a PPARγ agonist ligand.

## RESULTS

### TLR4 SNPs exacerbate colonic inflammation in DSS-treated mice

**Fig 1:**
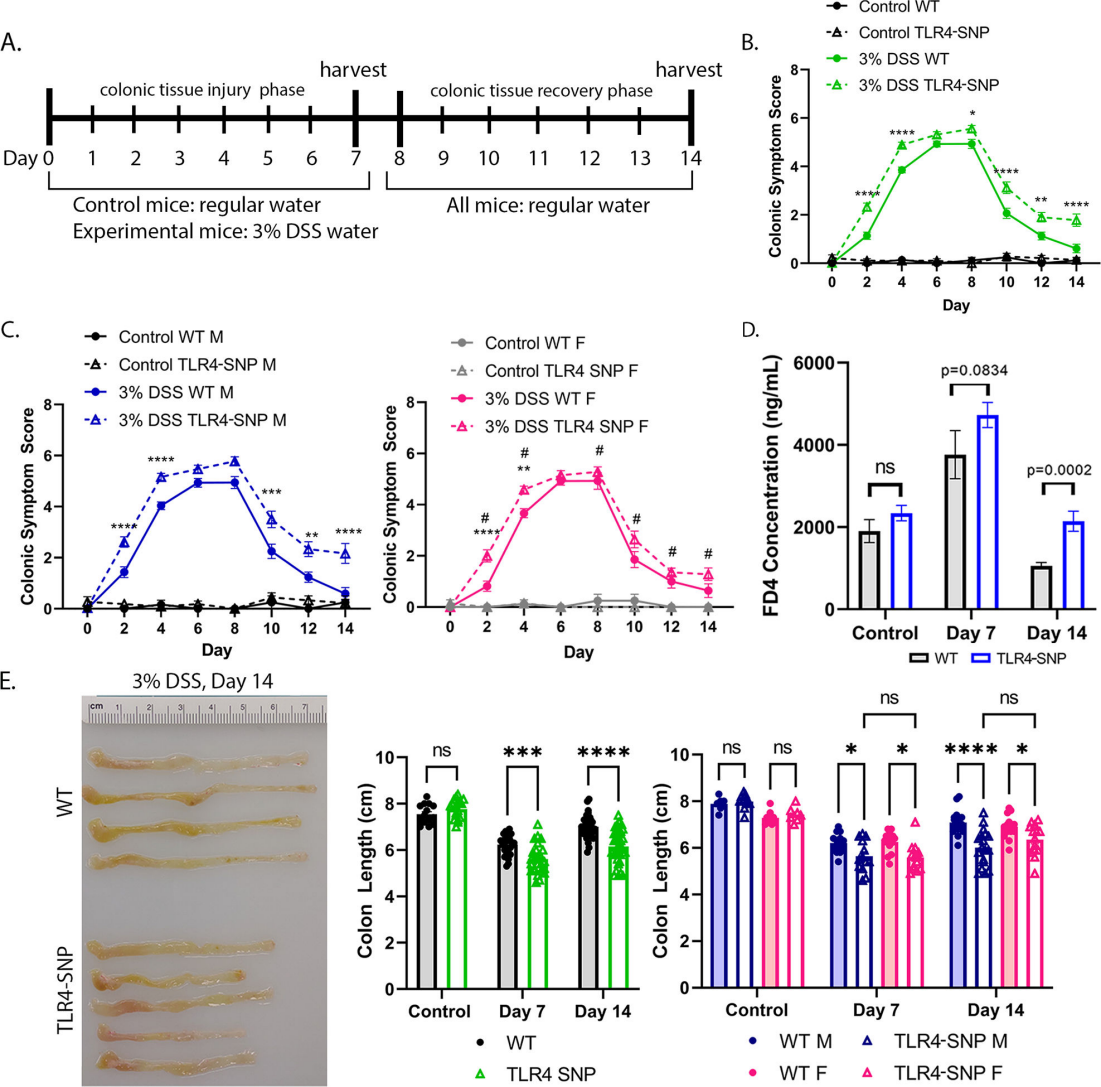
TLR4-SNP mice are more sensitive to DSS-induced colonic inflammation than C57BL/6J mice. (**A**) Schematic of the DSS experimental set-up. Experimental mice were given 3% DSS in their drinking water beginning on Day 0 and were switched to regular drinking water on Day 8, with fresh DSS water replaced on Days 2, 4, and 6. Control mice received regular drinking water throughout the experiment. On alternating days, stool consistency and fecal occult blood were assessed. Mice were euthanized and colons were resected on Day 7 or on Day 14. (**B**) Mean ± SEM colonic symptom scores (stool consistency score + fecal occult blood score) over time in control and DSS-treated WT (C57BL/6J) and TLR4-SNP mice (males and females combined). (**C**) Comparison of male only (left panel, blue graph) and female only (right panel, pink graph), control and DSS-treated, WT vs TLR4-SNP mouse responses. Two-way ANOVA with Sidak *post-hoc* multiple comparisons test. **P < 0.01, ***P < 0.001, ****P < 0.0001. On the female graph, # indicates days in which 3% DSS-treated female TLR4-SNP mice were determined to have significantly lower colonic symptom scores compared to 3% DSS-treated male TLR4-SNP mice (Student’s *t-*test). Results are derived from five independent experiments in which, together, control WT (*n* = 14; 6 males); control TLR4-SNP (*n* = 18; 11 males); 3% DSS WT Days 0–7 (*n* = 57; 30 males), Days 7–14 (*n* = 31; 17 males); 3% DSS TLR4-SNP Days 0–7 (*n* = 58; males), Days 7–14 (*n* = 32; 18 males). (**D**) Colonic permeability in WT vs TLR4-SNP male mice measured by the concentration of 4 kDa fluorescein isothiocyanate (FITC)-dextran in sera 4 h after administration of FITC-dextran. One-tailed Student’s *t-*test at each time point. Results are derived from four independent experiments in which, together, control WT (*n* = 10); control TLR4-SNP (*n* = 12); 3% DSS WT, Day 7 (*n* = 18), Day 14 (*n* = 15); 3% DSS TLR4-SNP Day 7 (*n* = 16), Day 14 (*n* = 17). (**E**) Representative colons from DSS-treated WT and TLR4-SNP mice at Day 14 (left panel). Mean colon lengths (showing individual responses) at Days 7 and 14 in control and DSS-treated WT and TLR4-SNP mice: males and females combined (center panel) and males vs females (right panel). Two-way ANOVA with Sidak (males and females combined) or Tukey (males vs females) *post-hoc* multiple comparisons test. *P < 0.05; **P < 0.01; ***P < 0.001; ****P < 0.0001. Results are derived from five independent experiments in which, together, control WT (*n* = 14; 6 males); control TLR4-SNP (*n* = 18; 11 males); 3% DSS WT, Day 7 (*n* = 26; 13 males), Day 14 (*n* = 31; 17 males); 3% DSS TLR4-SNP, Day 7 (*n* = 26; 13 males), Day 14 (*n* = 32; 18 males).

Previous studies reported that TLR4^−/−^ mice are more sensitive to DSS-induced colitis than WT mice (46, 47). In humans, the *TLR4* SNPs encoding D299G and T399I lead to LPS hyporesponsiveness (8) and are associated with IBD (10, 60 – 73). To better understand the role of TLR4 SNPs in IBD, we utilized “TLR4-SNP” knock-in mice that express two homologous point mutations in *Tlr4* (encoding D298G/N397I) and have been shown to result in LPS hyporesponsivness and related phenotypes (14). We hypothesized that mice expressing these homologous SNPs would exhibit a more severe disease phenotype upon DSS administration, a widely recognized model for chemically-induced colonic tissue inflammation, injury, and repair resembling human colitis (56, 74 – 78). Both WT C57BL/6J and TLR4-SNP mice (on a C57BL/6J background) received 3% DSS in their drinking water for 8 days *ad libitum* (“tissue injury phase,” Days 0–7), followed by 7 days of regular drinking water (“tissue recovery phase,” Days 8–14) (Fig. 1A). Control mice received regular drinking water only.

TLR4-SNP mice developed more severe colitis than WT mice throughout the experimental period as measured by “colonic symptom score” (Fig. 1B). When the responses of male and female TLR4-SNP mice were directly compared, male TLR4-SNP mice exhibited both enhanced symptom scores from Days 0 to 7 and a reduced capacity for recovery from Days 8 to 14 (Fig. 1C, left panel) compared to female TLR4-SNP mice (Fig. 1C, right panel; significant differences between males and females are indicated by #). Human IBD and DSS-induced colitis are associated with increased colonic permeability (55, 76, 79 – 86). Relative colonic permeability, assessed by detecting serum fluorescence in mice 4 h after oral administration of 4 kDa fluorescein isothiocyanate (FITC)-dextran, revealed no differences in permeability between control WT and TLR4-SNP mice (Fig. 1D). By Day 7, both strains exhibited increased serum FITC-dextran levels, with TLR4-SNP mice trending toward significantly greater colonic permeability compared to WT mice (Fig. 1D). By Day 14, WT and TLR4-SNP mice exhibited reduced serum levels of FITC-dextran compared to Day 7 levels; however, TLR4-SNP mice had significantly higher serum fluorescence than WT mice (Fig. 1D), suggesting that TLR4-SNP mice have an impaired ability to repair colonic damage. In addition, at Days 7 and 14, TLR4-SNP mice exhibited significantly shorter colon lengths, indicative of an increased inflammatory state, compared to WT mice (Fig. 1E, left and center panels); however, no sex differences in colon length were observed (Fig. 1E, right panel).

**Fig 2:**
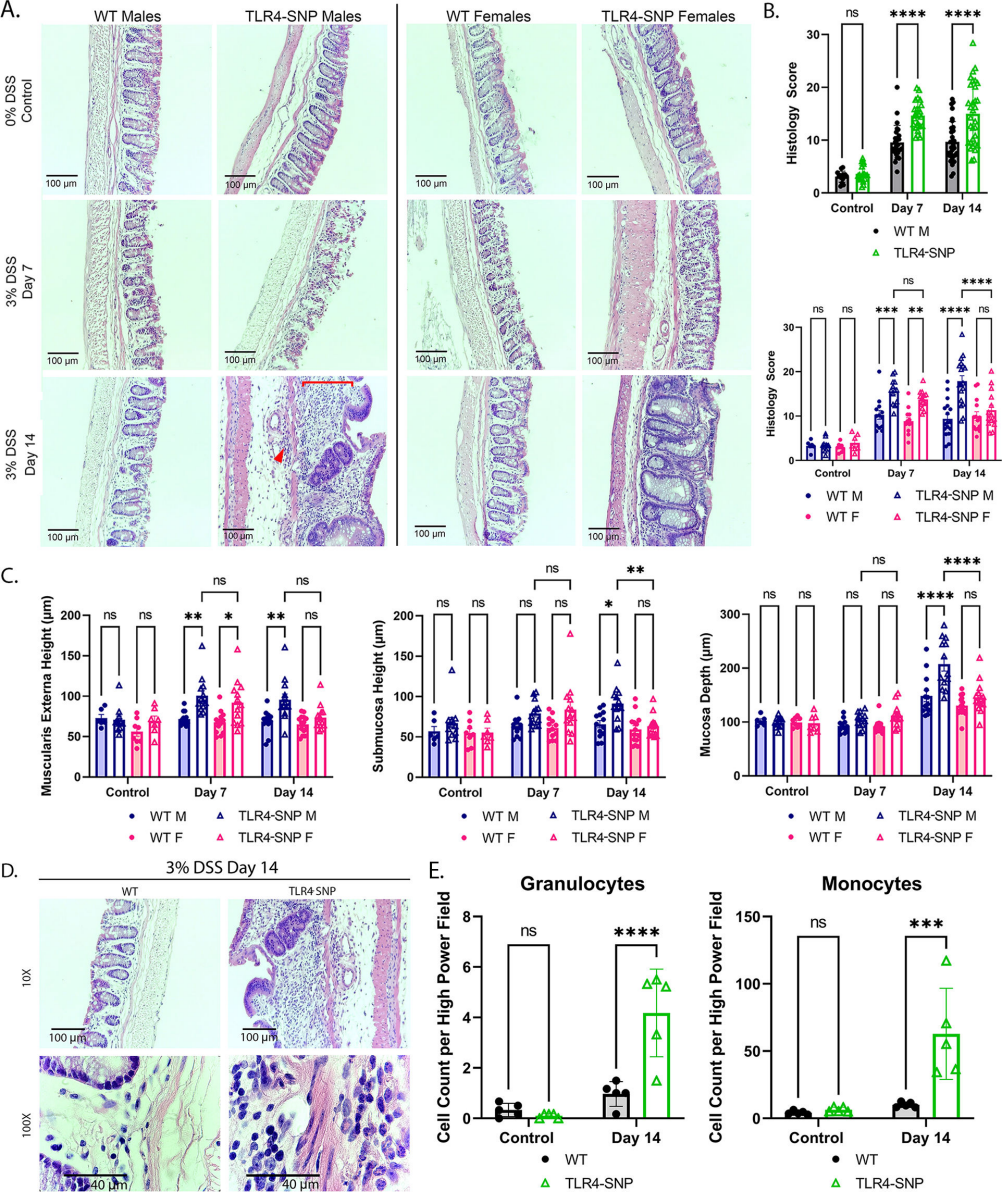
TLR4-SNP mice develop exacerbated colonic histopathology by Day 14. (**A**) Representative H&E-stained images of control and DSS-treated WT and TLR4-SNP distal 1 cm colon (DC) sections at Days 7 and 14 (males and females). In the TLR4-SNP Male Day 14 image, the red line indicates the region of extensive inflammatory cells in the mucosa; red arrowhead indicates inflammatory cells infiltrating into the submucosa. (**B**) Histology scores of H&E-stained DC sections from control and DSS-treated WT vs TLR4-SNP mice at Days 7 and 14 (showing individual responses): males and females combined (top panel) and males vs females (bottom panel). Two-way ANOVA with Sidak *post-hoc* multiple comparisons test. *P < 0.05; **P < 0.01; ***P < 0.001; ****P < 0.0001. Results were derived from five independent experiments in which, together, control WT (*n* = 14; 6 males); control TLR4-SNP (*n* = 18; 11 males); 3% DSS WT, Day 7 (*n* = 26; 13 males), Day 14 (*n* = 31; 17 males); 3% DSS TLR4-SNP, Day 7 (*n* = 26; 13 males), Day 14 (*n* = 32; 18 males). (**C**) Measurements of histologic region sizes (mucosa depth, submucosal height, muscularis externa height) from control and experimental H&E-stained DC sections at Days 7 and 14. Two-way ANOVA with Tukey’s *post-hoc* multiple comparisons test. *P < 0.05; **P < 0.01; ****P < 0.0001. Results are derived from five independent experiments in which, together, control WT (*n* = 14; 6 males); control TLR4-SNP (*n* = 18; 11 males); 3% DSS WT Day 7 (*n* = 26; 13 males), Day 14 (*n* = 27; 13 males); 3% DSS TLR4-SNP Day 7 (*n* = 26; 13 males), Day 14 (*n* = 27; 13 males). (**D**) Additional microscopic images were taken of the identical tissue sections shown in Fig. 2A (DSS, male WT vs TLR4-SNP, Day 14) at both 10× and 100× magnification to better illustrate the observed inflammatory differences between the two mouse strains and to allow for quantification of the infiltrating cell types as shown in Fig. 2E. (**E**) Number of granulocytes (left panel) and monocytes (right panel) in 100× high power H&E-stained DC sections of WT and TLR4-SNP mice. Two-way ANOVA with Sidak’s *post-hoc* multiple comparisons test. ***P < 0.001; ****P < 0.0001. Data are derived from two independent experiments, *n* = 5 mice/strain for each time point.

Disease severity was also assessed in H&E-stained micrographs of the distal 1 cm colon (DC) of WT and TLR4-SNP mice. No significant differences were observed between untreated WT and TLR4-SNP mice (Fig. 2A, top row). On Day 7, DSS-treated WT mice exhibited few inflammatory infiltrates in the mucosa and submucosal regions compared to non-DSS-treated control mice and the crypt structure remained largely intact (Fig. 2A, middle row). In contrast, by Day 7, DSS-treated TLR4-SNP mice displayed disrupted crypt structures with inflammatory infiltrates in the mucosa and submucosal regions, accompanied by epithelial damage (Fig. 2A, middle row). By Day 14, the crypt structures of DSS-treated TLR4-SNP mice were severely disrupted by the extensive inflammatory infiltrates in the mucosa (Fig. 2A, bottom row; red line) and submucosal (Fig. 2A, bottom row; red arrowhead) regions in contrast to WT mice. These histopathological differences between WT and TLR4-SNP mice were more pronounced in male mice (Fig. 2A, left panels) and confirmed by histologic scoring (Fig. 2B) and measurements of the muscularis externa, submucosal height, and mucosal depth (Fig. 2C). The notable colonic shortening in DSS-treated TLR4-SNP mice that was observed grossly at Day 14 (Fig. 1E) correlated with broadening of the muscularis mucosa (Fig. 2C, left panel). Moreover, inflammatory infiltrates accumulated and contributed to edema in the submucosa (Fig. 2A, bottom row) which correlated with increased submucosal height (Fig. 2C, center panel), while observed crypt regeneration in response to damage was evidenced by increased mucosal depth (Fig. 2C, right panel). At higher magnification, Day 14 DSS-treated TLR4-SNP mice exhibited substantially greater cellular infiltration into the colonic mucosa and submucosa than WT mice (Fig. 2D), with infiltrating cells that were predominantly monocytic (Fig. 2E). Collectively, our observations of worse colonic symptoms, reduced colon lengths, and more severe histopathology in TLR4-SNP mice indicate an impaired ability to recover from DSS-induced colonic injury. These findings prompted us to investigate possible mechanism(s) underlying the lack of recovery in the TLR4-SNP mice.

### IL-4Rα-dependent signaling is necessary for tissue recovery from DSS-induced colonic damage

There are many studies that indicate that the microbiome is important in DSS-induced colitis (87 – 92). However, we initially carried out co-housing experiments in which WT and TLR4-SNP mice were co-housed for 3 weeks prior to DSS treatment and throughout the entire experiment to normalize their microbiomes (93, 94). Nonetheless, the TLR4-SNP mice remained much more sensitive to DSS (data not shown), indicating that the increased sensitivity of TLR4-SNP mice to DSS was not likely attributable to dysbiosis.

There is also substantial indirect evidence in the literature that anti-inflammatory M2-like Mφ contribute to resolution of DSS-induced colitis (40, 95 – 110). For example, adoptive transfer of *in vitro*-derived IL-4/IL-13-stimulated M2a Mφ into mice depleted of resident Mφ resulted in reduced dinitrobenzene sulfonic acid (DNBS)-induced colitis symptoms in mice (110). While M2 Mφ subsets are defined by their various inducers and anti-inflammatory products produced (111 – 114), we next focused our studies on the potential role of M2a Mφ since the only known inducers of this subset are IL-4 and IL-13 via signaling through a shared IL-4Rα chain (115, 116). IL-4Rα^−/−^ mice fail to generate significant M2a Mφ in response to IL-4 or IL-13 (117). For this reason, we sought direct evidence for a role for M2a Mφ in recovery from DSS-induced colitis by comparing the sensitivity of WT (BALB/c) and IL-4Rα^−/−^ (on a BALB/c background) mice to DSS.

**Fig 3:**
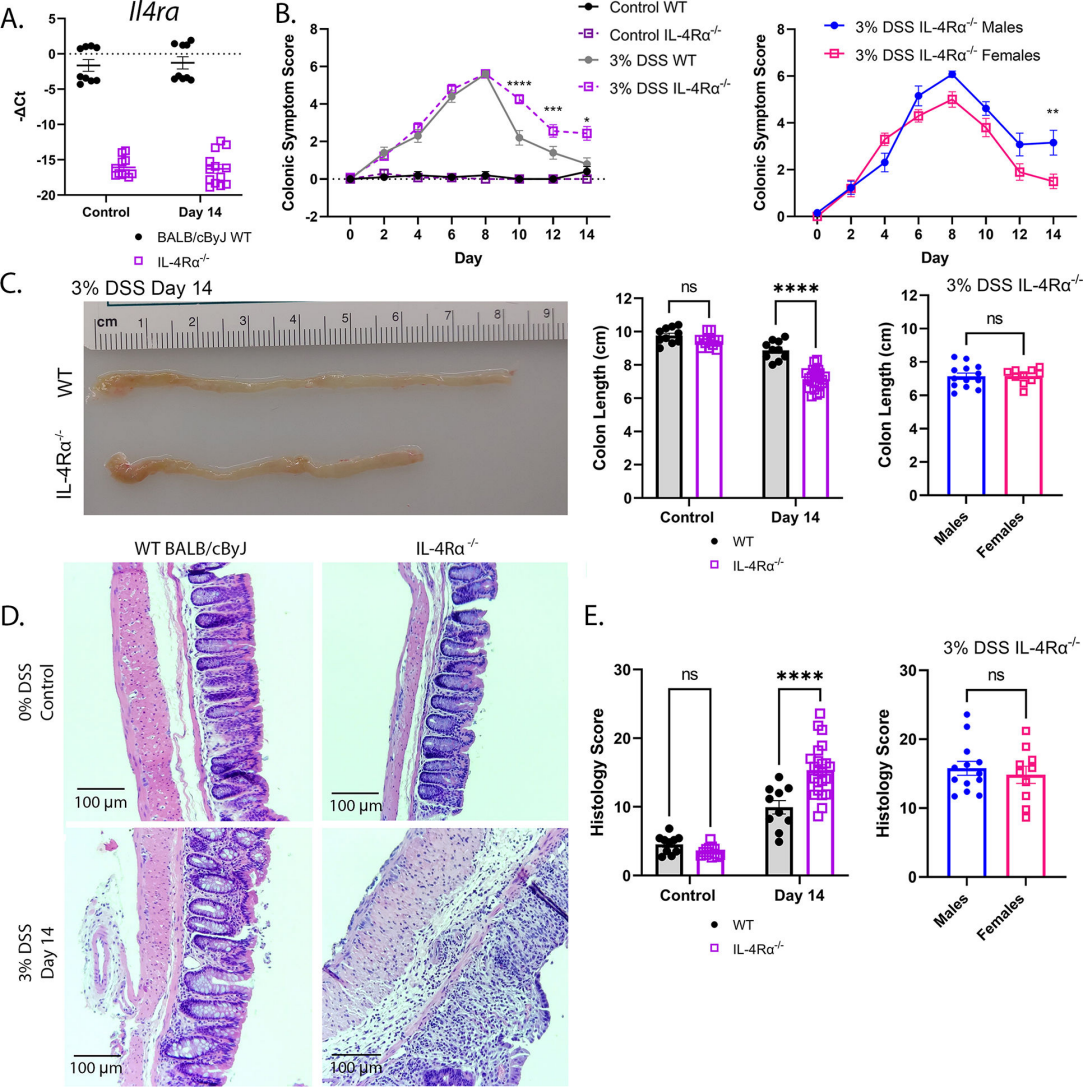
IL-4Rα^−/−^ mice are more sensitive to DSS-induced colitis than WT BALB/cByJ WT mice. (**A**) qRT-PCR was carried out on MC sections for *Il4ra* gene expression in control and DSS-treated WT and IL-4Rα^−/−^ mice to confirm genotype. (**B**) Colonic symptom scores (mean ± SEM) over time in control and DSS-treated WT vs IL-4Rα^−/−^ mice (left panel, responses of males and females mice combined; right panel, comparison of male vs female responses to DSS). Two-way ANOVA with Sidak’s *post-hoc* multiple comparisons test. *P < 0.05; **P < 0.01; ***P < 0.001; ****P < 0001. (**C**) Colon lengths at Day 14 in control and experimental WT vs IL-4Rα^−/−^ mice. Far left panel, representative DSS-treated WT and IL-4Rα^−/−^ colons at Day 14. Middle panel, male and female responses combined. Two-way ANOVA with Sidak *post-hoc* multiple comparisons test. Far right panel, DSS-treated male vs female IL-4Rα^−/−^ mice. Unpaired two-tailed Student’s *t-*test. (**D**) Representative H&E-stained images of DC sections from control and DSS-treated WT and IL-4Rα^−/−^ mice at Day 14. (**E**) Histology scores of H&E-stained DC sections from control and DSS-treated WT and IL-4Rα^−/−^ mice at Day 14 (left panel, males and females combined; right panel, male vs female, DSS-treated IL-4Rα^−/−^ mice). Two-way ANOVA with Sidak *post-hoc* multiple comparisons test. *P < 0.05; **P < 0.01; ***P < 0.001; ****P < 0.0001. Right panel: Histology scores at Day 14 in male vs female DSS-treated IL-4Rα^−/−^ mice. Unpaired two-tailed Student’s *t-*test. Results are derived from three independent experiments in which, together, control WT (*n* = 10; 5 males), control IL-4Rα^−/−^ (*n* = 13; 5 males); 3% DSS WT Day 14 (*n* = 10; 5 males); 3% DSS IL-4Rα^−/−^ Day 14 (*n* = 23; 13 males).

We first confirmed that IL-4Rα^−/−^ mice do not express IL-4Rα (Fig. 3A) by measuring *Il4ra* mRNA in colons of WT vs IL-4Rα^−/−^ mice. WT mice displayed unaltered *Il4ra* mRNA levels in response to DSS treatment, while *Il4ra* mRNA levels in IL-4Rα^−/−^ colons were at or below the limit of detection. Following the tissue injury phase, IL-4Rα^−/−^ mice exhibited an impaired ability to recover from DSS-induced colonic injury compared to WT mice evidenced by statistically increased colonic symptom scores beginning at Day 10 (Fig. 3B, left panel) with males being more sensitive than females (Fig. 3B, right panel). By Day 14, the IL-4Rα^−/−^ mice exhibited significantly shorter colons than WT mice (Fig. 3C) and increased pathology (Fig. 3D), which was confirmed by histologic scoring (Fig. 3E). Few inflammatory infiltrates were seen in the mucosal and submucosal regions in DSS-treated WT mice at Day 14 compared to non-DSS treated controls and the crypt structure remained intact in contrast to the IL-4Rα^−/−^ mice (Fig. 3D). At Day 14, DSS-treated IL-4Rα^−/−^ mice exhibited visibly disrupted crypts, with abundant inflammatory infiltrates in the mucosal and submucosal regions and extensive epithelial damage (Fig. 3D). No sex differences were observed for colon length or histology scores (Fig. 3C and E). These findings are the first to directly support the conclusion that IL-4- and/or IL-13-induced signaling via IL-4Rα is necessary for tissue recovery from DSS-induced damage.

### TLR4-SNP mice exhibit a reduced capacity to induce M2a Mφ in response to DSS

**Fig 4:**
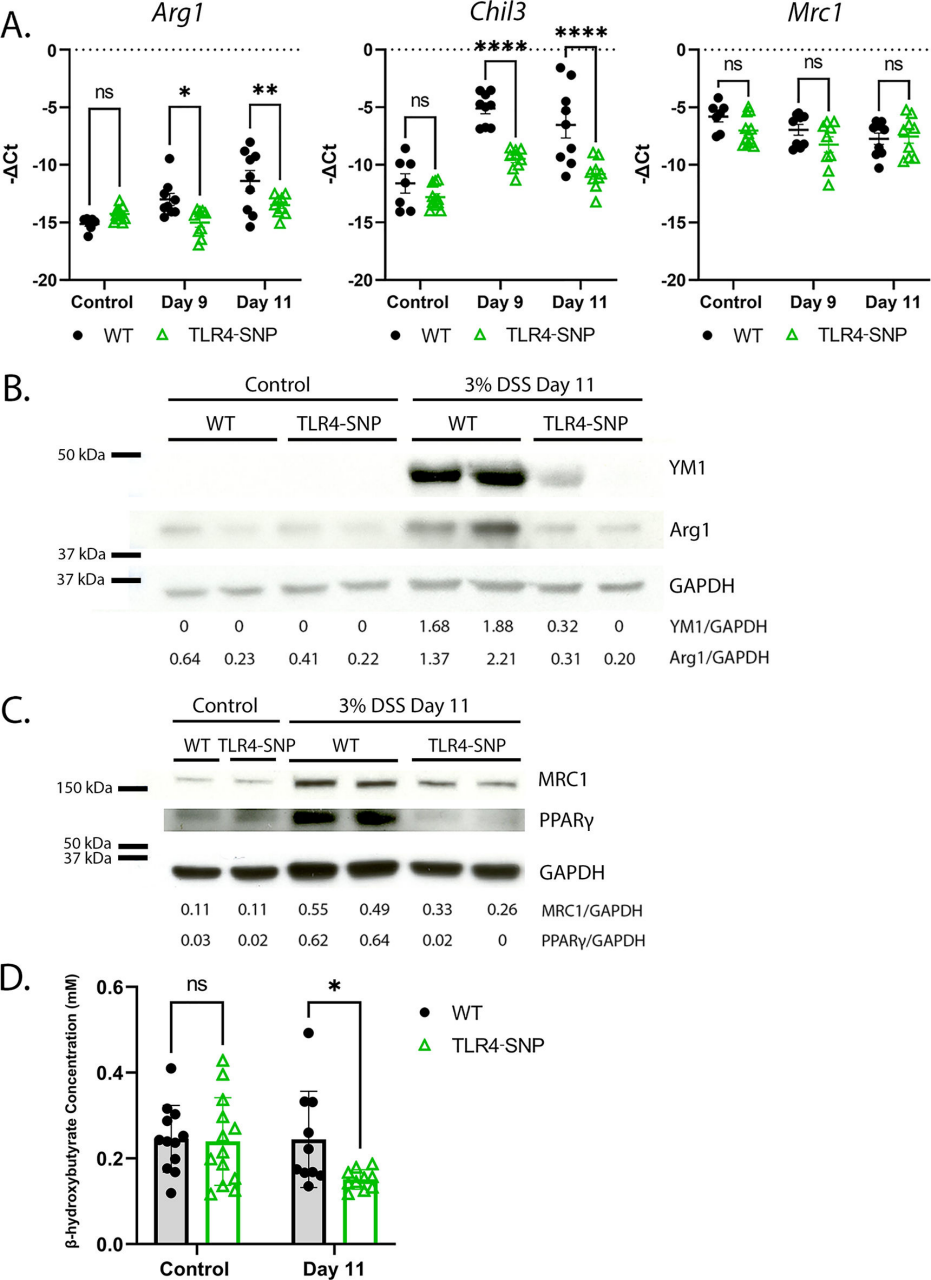
DSS-treated TLR4-SNP mice exhibit reduced M2a markers in the colon and reduced β-hydroxybutyrate (β-HB) levels in the sera. (**A**) M2a gene expression measured in the MC of male WT vs TLR4-SNP mice, comparing control, Day 9, and Day 11 samples by qRT-PCR. One-way ANOVA with Sidak multiple comparisons test. *P < 0.05; **P < 0.01; ****P < 0.0001. Data are derived from two independent experiments. In total, control WT *n* = 11; control TLR4-SNP *n* = 15; WT Day 9 *n* = 13, Day 11 *n* = 13; TLR4-SNP Day 9 *n* = 15, Day 11 *n* = 15. (**B**) M2a (Ym1, Arg1) protein production measured in DC samples in WT vs TLR4-SNP mice, comparing control and Day 11 samples. Data are representative of results from two independent experiments totaling control WT *n* = 5; control TLR4-SNP *n* = 5; WT Day 11 *n* = 9; TLR4-SNP Day 11 *n* = 9. (**C**) M2a (Mrc1, PPARγ) protein production measured in DC samples in WT vs TLR4-SNP mice, comparing control and Day 11 samples. Data representative of results from two independent experiments totaling control WT *n* = 5; control TLR4-SNP *n* = 5; WT Day 11 *n* = 9; TLR4-SNP Day 11 *n* = 9. (**D**) β-HB concentration measured in the sera of control and DSS-treated, WT vs TLR4-SNP male mice at Days 9 and 11. Two-way ANOVA with Sidak multiple comparisons. *P < 0.05; **P < 0.01; ***P < 0.001; ****P < 0.0001. Data are derived from three independent experiments in which, together, control WT *n* = 12; control TLR4-SNP *n* = 13; WT Day 11 *n* = 10; TLR4-SNP Day 11 *n* = 10.

Since IL-4Rα signaling was required for the repair of DSS-induced colitis, we next sought to determine if the worsened colitis of DSS-treated TLR4-SNP mice was attributable to a decreased capacity for differentiation of M2a Mφ. Since the repair phase occurs between Days 8 and 14, Days 9 and 11 were selected to measure the induction of M2a Mφ markers in the colons of WT and TLR4-SNP mice. We compared the induction of M2a genes in colon sections of control and DSS-treated mice. M2a Mφ markers, *Arg1,* and *Chil3* mRNA levels were significantly reduced in TLR4-SNP colons at Days 9 and 11, while *Mrc1* mRNA showed a trend toward reduced M2a gene expression in TLR4-SNP colon sections compared to WT mice, particularly at Day 9 (Fig. 4A). These observations were confirmed by Western analysis showing reduced Arginase 1 (Arg1), Ym1 (Chil3) (Fig. 4B), and Mrc1 (Fig. 4C) protein levels in TLR4-SNP colon homogenates at Day 11. These findings indicate that TLR4-SNP mice have an impaired ability to induce M2a Mφ markers in response to DSS.

### Reduced circulating levels of β-hydroxybutyrate in TLR4-SNP mice

β-Hydroxybutyrate (β-HB) is a metabolite produced by both animals and bacteria (118 – 120) and a hallmark of Gram-negative bacteria (121 – 124). Numerous studies have implicated the microbiome in IBD pathogenesis (87 – 92), noting significantly reduced β-HB levels in the colonic mucosa (103) and sera (125, 126) of patients with IBD. Introduction of bacteria engineered to produce β-HB (127) or intrarectal administration of β-HB (103) improved DSS-induced symptoms in mice. *In vitro*, Huang et al. showed that β-HB synergistically enhanced IL-4-induced signaling and, *in vivo*, exogenous administration of β-HB to WT mice induced the M2a genes *Arg1* and *Chil3* (103). Therefore, we hypothesized that the observed defect in repair in the TLR4-SNP mice could be attributable, in part, to impaired M2a Mφ differentiation driven by β-HB. In sera of DSS-treated TLR4-SNP mice, β-HB levels were significantly reduced at Day 11 compared to WT mice (Fig. 4D).

### TLR4-SNP mice produce reduced levels of PPARγ in response to DSS

Previous studies reported that differentiation of M2a Mφ is dependent on the ligand-activated transcription factor, PPARγ (128 – 138). PPARγ is a nuclear hormone receptor (NHR) that forms a heterodimer complex with another NHR, retinoid X receptor (RXR), to regulate the expression of various adipocyte-specific and M2a Mφ differentiation genes (128 – 130, 139, 140). PPARγ is highly expressed in adipose tissue and the colon (141), particularly in epithelial cells (142) and Mφ (143). Rectal biopsies from patients with active UC show reduced PPARγ mRNA and protein levels in the colonic mucosa (144, 145). Animal models of IBD revealed that PPARγ influences disease timing and severity (146). Consistent with reduced levels of Ym1 (Chil3), Arg1, and Mrc1 proteins in colon homogenates (Fig. 4B and C), DSS-treated TLR4-SNP mice also produced lower levels of PPARγ protein when compared to WT mice (Fig. 4C).

### The PPARγ agonist ligand, rosiglitazone, improved DSS-induced colitis in TLR4-SNP mice therapeutically

**Fig 5:**
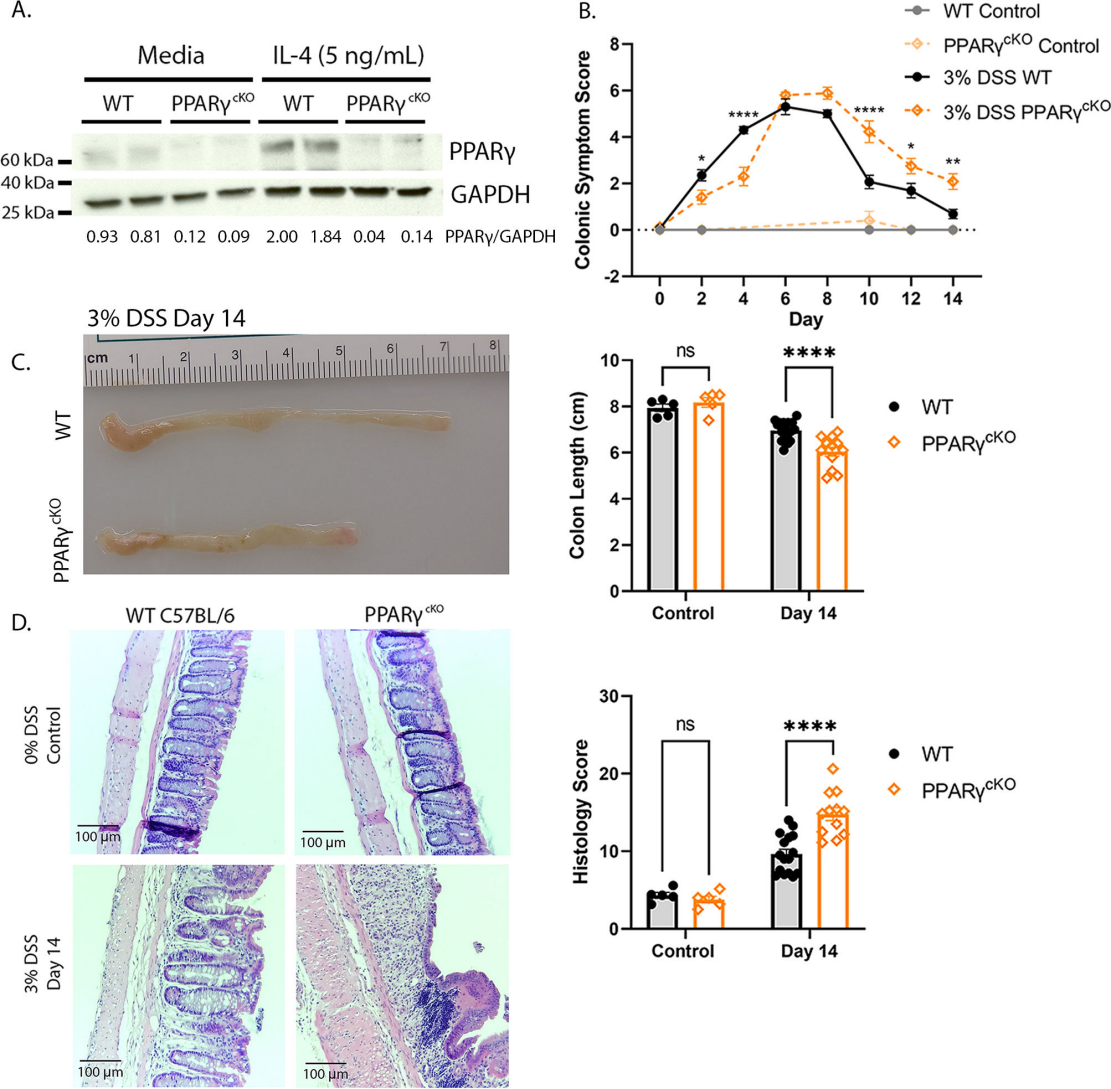
PPARγ^cKO^ mice are more sensitive to DSS-induced colitis than WT mice. (**A**) PPARγ protein expression in thioglycollate-elicited macrophages from WT and PPARγ^cKO^ mice in the absence or presence of IL-4 (24 h) to confirm genotype. (**B**) Colonic symptom scores (Mean ± SEM) in control and DSS-treated WT vs PPARγ^cKO^ mice (responses of male and female mice combined). Two-way ANOVA with Sidak’s *post-hoc* multiple comparisons test. *P < 0.05; **P < 0.01; ***P < 0.001; ****P < 0001. (**C**) Representative colon lengths at Day 14 in control and DSS-treated WT vs PPARγ^cKO^ mice. Left panel, gross image of representative DSS-treated WT and PPARγ^cKO^ colons on Day 14. Right panel, male and female responses combined. Two-way ANOVA with Sidak’s *post-hoc* multiple comparisons test. ****P < 0001. (**D**) Left panel: representative H&E-stained images of DC sections. Right panel: histology scores of H&E-stained DC sections from control and DSS-treated WT and PPARγ^cKO^ mice on Day 14, males and females combined. Two-way ANOVA with Sidak *post-hoc* multiple comparisons test. ****P < 0001. Results were derived from three independent experiments in which in total control WT (*n* = 5), control PPARγ^cKO^ (*n* = 5), 3% DSS WT (*n* = 16 males), 3% DSS PPARγ^cKO^ (*n* = 12; 8 males). Although the early kinetics of DSS-induced disease were delayed in PPARγ^cKO^ compared to WT mice, we observed deaths in four males and one female, suggesting that myeloid PPARγ plays a key role in tissue repair.

To confirm the role of PPARγ in DSS-induced colitis, we utilized PPARγ conditional knockout (PPARγ^cKO^) mice originally derived by crossing homozygous floxed PPARγ mice with a transgenic mouse containing the *Cre* recombinase gene under the control of the murine Lysozyme M (*Lyz2*) promoter to delete the *Pparg* gene in lysozyme-producing cells (i.e., predominantly Mφ and neutrophils) (133, 147 – 149). A lack of PPARγ protein expression in medium- or IL-4-stimulated, PPARγ^cKO^ vs WT thioglycollate-elicited peritoneal Mφ was confirmed by Western blot (Fig. 5A). Although DSS-treated PPARγ^cKO^ mice presented on Days 2 and 4 with milder colonic symptoms compared to DSS-treated WT mice, their disease severity significantly increased between Days 10 and 14, suggesting an impaired ability to recover (Fig. 5B). By Day 14, PPARγ^cKO^ mice exhibited significantly shorter colon lengths (Fig. 5C) and increased histopathology (Fig. 5D) than WT mice, phenotypes also observed in DSS-treated TLR4-SNP and IL-4Rα^−/−^ mice. No sex differences were observed for any of the parameters measured.

**Fig 6:**
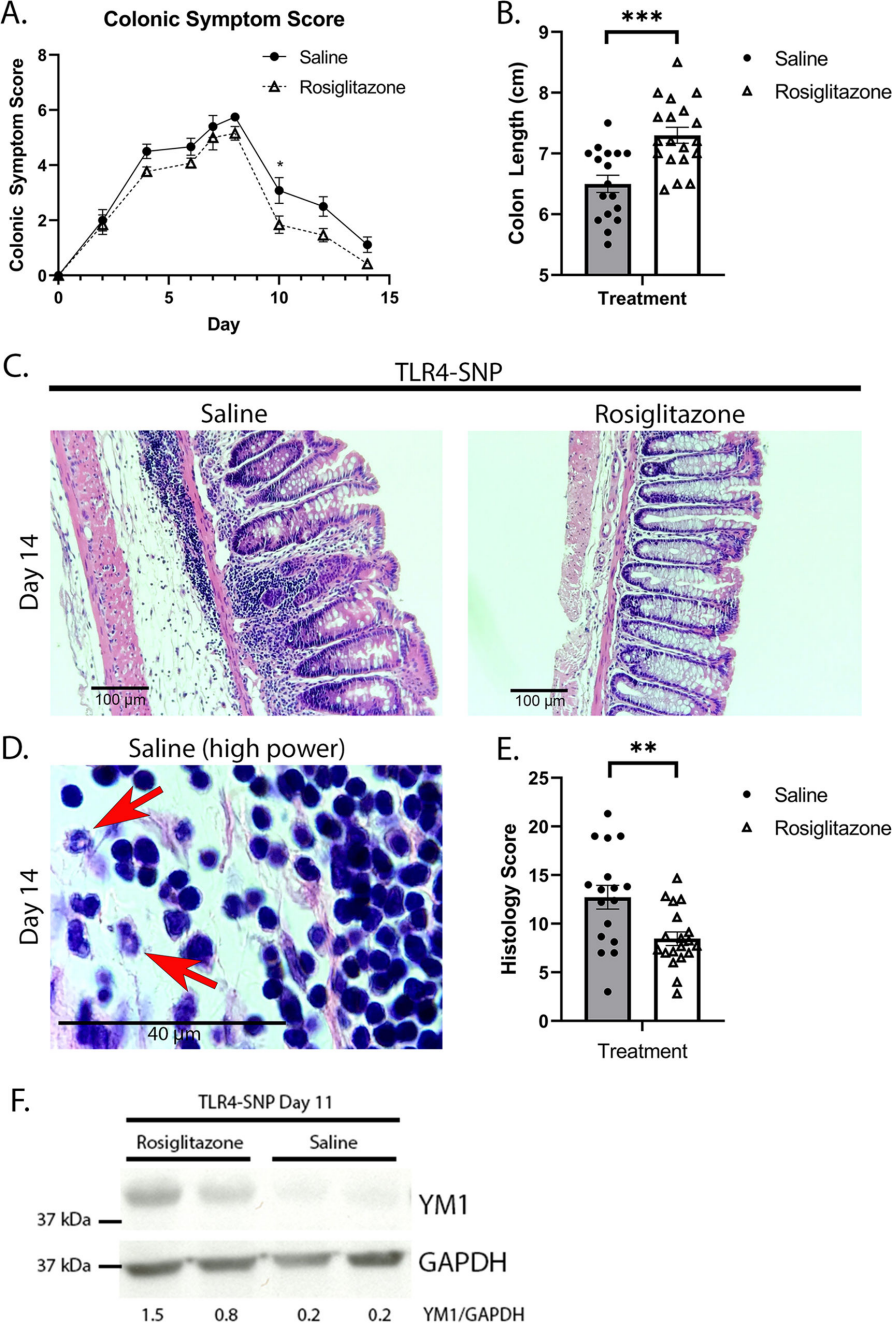
Administration of rosiglitazone, a PPARγ agonist ligand, ameliorates DSS-induced colonic damage in TLR4-SNP mice. Male TLR4-SNP mice were administered DSS starting on Day 0. On Days 2–7, mice were administered either saline or rosiglitazone (25 mg/kg) once daily i.p. (**A**) Average colonic symptom score over time in saline- and rosiglitazone-treated male TLR4-SNP mice. Two-way ANOVA with Sidak’s multiple comparisons; *P < 0.05. (**B**) Colon lengths at Day 14 in saline- and rosiglitazone-treated TLR4-SNP mice. Unpaired two-tailed Students *t-*test. ***P < 0.001. (**C**) Representative H&E-stained images of DC sections from saline- and rosiglitazone-treated TLR4-SNP mice at Day 14. (**D**) High-power image of submucosal infiltrating inflammatory cells in a representative DSS- and saline-treated TLR4-SNP mouse on Day 14. The infiltrating cells are predominantly mononuclear with occasional granulocytic cells (red arrows). (**E**) Right panel: histology scores of H&E-stained DC sections from saline- and rosiglitazone-treated TLR4-SNP mice at Day 14. Unpaired two-tailed Students *t-*test; **P < 0.01. Results were derived from three independent experiments. In total, TLR4-SNP saline *n* = 17; TLR4-SNP rosiglitazone *n* = 19. (**F**) Ym1 (Chil3) protein expression at Day 11 in DC of DSS-treated TLR4-SNP mice therapeutically administered rosiglitazone or saline. Data are representative of results from two independent experiments totaling TLR4-SNP rosiglitazone-treated *n* = 9; TLR4-SNP saline-treated *n* = 8.

Thiazolidinedione (TZD) agents, such as rosiglitazone, are PPARγ agonist ligands shown previously to ameliorate DSS-induced colitis in mice (139, 150 – 154) and 2,4,6-trinitrobenzene sulfonic acid (TNBS)-induced colitis in rats (155). TLR4-SNP mice were administered 3% DSS in their drinking water starting on Day 0 and then treated therapeutically with saline or rosiglitazone (once daily from Day 2 to Day 7). On Days 8 to 14, all mice were given regular drinking water. Compared to DSS + saline-treated TLR4-SNP mice, TLR4-SNP mice treated with DSS + rosiglitazone displayed reduced clinical symptoms (Fig. 6A), increased colon lengths at Day 14 (Fig. 6B), repaired crypt structure, and reduced numbers of infiltrating inflammatory cells (Fig. 6C). Under high power, the infiltrating cells in the DSS + saline-treated TLR4-SNP mice were predominantly mononuclear with a few granulocytic cells (indicated with red arrows; Fig. 6D). Histopathological changes were reflected in the histology scores (Fig. 6E). Consistent with a role for M2a Mϕ in the resolution of DSS-induced colitis, the rosiglitazone-treated TLR4-SNP mice exhibited increased expression of the M2a protein, Ym1 (Chil3), compared to the saline-treated group (Fig. 6F). These findings indicate that therapeutic treatment with the PPARγ agonist ligand improves DSS-induced colitis symptoms in the hypersensitive TLR4-SNP mice.

## DISCUSSION

The D299G and T399I substitutions in human TLR4 have been shown to exacerbate or ameliorate inflammation in different diseases and disease models, including infection, inflammation, and cancer (3, 58, 156). In IBD, the majority of independent association studies and meta-analyses reported an association between inheritance of human *TLR4* SNPs encoding D299G/T399I and increased risk of IBD in humans (10, 60 – 70); however, some studies with smaller sample sizes reported no significant association (65 – 67, 71 – 73). Early studies showed that mice with natural or engineered deficiencies in TLR4 signaling (e.g., C3H/HeJ, C57BL/10ScN, TLR4^−/−^) exhibited increased susceptibility to chemically-induced colitis (43, 46, 47). Rakoff-Nahoum et al. attributed the increased mortality of MyD88^−/−^ mice and TLR4^−/−^ mice to increased colonic bleeding resulting from epithelial denudation and glandular mucodepletion (46). The mechanism(s) by which reduced TLR4 expression contributes to colitis sensitivity are clearly multifaceted and still not entirely understood. While commensal overgrowth and hyperinfiltrating leukocytes were ruled out as potential contributors to disease, dysregulated epithelial cell proliferation and differentiation and compromised expression of cytoprotective and repair factors like IL-6, TNF, KC-1, and heat shock proteins were identified as potential contributing factors (46). The presence of the microbiome was required for cytokine production by the colon and recognition of commensal microbial ligands by TLRs was essential for protection from DSS-induced epithelial injury and mortality (46). For this reason, it is surprising that targeting of TLR4 (157) early during DSS treatment by administration of a monoclonal antibody directed against the TLR4-MD-2 complex (158) or a synthetic TLR4 antagonist (159) reduced disease. In contrast, Fukata et al. reported increased bacterial translocation in DSS-treated TLR4^−/−^ mice and reduced neutrophil recruitment due to impaired MIP-2 production by TLR4-deficient Mφ (47).

Since the human D299G/T399I mutations in TLR4 are co-inherited by a significant proportion of individuals (33), “TLR4-SNP” mice were engineered to enable mechanistic studies by which these TLR4 SNPs impact diseases. To date, these mice have been shown to reflect phenotypes in humans, including LPS hyporesponsiveness, increased sensitivity to Gram negative infection and RSV, and increased resistance to influenza infection (14). In addition, the TLR4-SNP mice exhibit reduced allergic inflammation with LPS and OVA but increased inflammation, eosinophilic infiltration, and Th2 cytokines following house dust mite allergen induction, reflecting a significant effect of the environment on their Th2-dependent responses (160). Further, TLR4-SNP mice show reduced antibody responses and a deficiency in class switching (also Th2-dependent) in response to immunization to T-dependent antigen when adjuvanted with a lipid A-based adjuvant (161).

Although DSS is widely used to induce colitic disease in mice, we recognize that this model has limitations (162). DSS is toxic to epithelial cells resulting in loss of barrier integrity by redistribution of the tight junction components, increased permeability, increased epithelial apoptosis and proinflammatory cytokines, and entry of luminal antigens and microorganisms that induce inflammation (162). The pathogenesis of IBD is complex, and many mechanisms have been implicated (51 – 53). In healthy individuals, multiple cellular programs coordinate to effectively promote healing of damaged tissues. One cell type that is particularly important for healing is the alternatively activated or repair “M2” Mφ.

Mφ mediate innate immune responses to PAMPs and DAMPs and play a key role in tissue repair and regeneration (163 – 165). The presence of cytokines and other environmental signals promotes Mφ polarization to a variety of phenotypes. Mφ are often described in terms of a “differentiation spectrum,” with “classically activated” “M1” Mφ, induced by potent inflammatory stimuli such as LPS and IFN-γ, at one end of the differentiation spectrum, and “alternatively activated” or “tissue repair” (166) “M2” Mφ at the other end of the spectrum (113). While M2 Mφ differentiation was originally attributed to signaling by IL-4 and IL-13 through a shared IL-4Rα chain to activate M2-associated genes (116, 166), the definition of “M2” Mφ is now far more nuanced and subsets have been defined (M2a, M2b, M2c, M2d, regulatory) based on distinct inducing agents and transcriptomic patterns (112, 114).

By using IL-4Rα^−/−^ mice, our study has provided the first direct experimental evidence for the role of IL-4/13 signaling in the repair of DSS-induced colitis in WT mice (Fig. 3). IL-4 and IL-13 signaling through a shared receptor, IL-4Rα, leads to activation of specific transcription factors, e.g., STAT-6, EGR-2 (112, 167), that, in turn, recruit the transcription factor PPARγ to activate promoters of specific “M2a” genes (112, 130, 167, 168). We extended our findings in the IL-4Rα^−/−^ mice by showing that mice in which PPARγ had been deleted in myeloid cells (133, 147 – 149) were also extremely sensitive to DSS-induced colitis (Fig. 5). We recognize that LysMCre deletion is not absolute and that PPARγ on other cell types (e.g., epithelial cells) remains functional and may even compensate to some degree for the lack of PPARγ on myeloid cells. Nonetheless, like DSS-treated IL-4Rα^−/−^ and TLR4-SNP mice, the PPARγ^cKO^ mice phenocopy the impaired repair responses to DSS. When induction of M2a-related mRNA and protein species were measured at Days 9 and 11, colons of DSS-treated TLR4-SNP mice exhibited reduced levels of M2a Mφ RNA and proteins, including PPARγ protein (Fig. 4A through C). These findings are consistent with previous studies showing that in contrast to WT Mφ TLR4^−/−^ Mφ failed to induce *Pparg* mRNA in response to either IL-4 stimulation or RSV infection (135). Treatment of male and female, WT and TLR4-SNP thioglycollate-induced, peritoneal Mφ with exogenous IL-4 resulted in no significant difference in the levels of *Arg1* and *Chil3* mRNA, indicating that TLR4-SNP Mφ do not have an intrinsic defect in the ability to respond through IL-4Rα (data not shown).

PPARγ heterodimerizes with the NHR, RXR, to activate transcription of many M2a genes (e.g., *Arg1, Mrc1, Chil3*) (112, 130, 167), and transcriptional activity can be further modulated by endogenous and exogenous agonist ligands that bind to each component of the heterodimer (169). Many studies have investigated the therapeutic effect of ligand activation of the PPARs in IBD (141, 150, 170 – 173), including human clinical trials (174 – 176) and studies (177). TZDs are a class of structurally related PPARγ ligands originally designed as antidiabetic drugs. In particular, rosiglitazone acts as a highly specific ligand agonist of PPARγ (178, 179) that enhances M2a Mφ polarization (130, 180, 181), reduces DSS-induced inflammation *in vivo* (150), and leads to overall anti-inflammatory effects (182). Rosiglitazone showed promise in a clinical trial for treatment of active UC (175) but is no longer prescribed due to cardiotoxicity (183). Rosiglitazone therapy resolved lung histology and enhanced the expression of M2a Mφ in rodent models of RSV-induced lung injury (135). Another structurally related TZD, pioglitazone, showed similar outcomes in influenza infection (184). We hypothesized that therapeutic administration of rosiglitazone would also contribute to the resolution of DSS-induced colitis in the TLR4-SNP mice. Indeed, rosiglitazone effectively reversed DSS-induced colonic damage in the TLR4-SNP mice (Fig. 6), further supporting the conclusion that PPARγ may be an effective therapeutic target in patients that express the D299G and T399I TLR4 SNPs. Importantly, recent studies suggest that it is possible to develop PPARγ ligands that dissociate one cell-specific PPARγ function from another (185), offering the possibility of being able to identify a new PPARγ agonist ligand that will ameliorate IBD but not exert cardiotoxicity.

Since the microbiome has been strongly implicated in IBD pathogenesis, future studies will be required to determine whether the microbiomes of WT and TLR4-SNP mice differ and whether potential microbial differences affect the observed phenotypic responses to DSS in the two strains. Since β-HB is a metabolite associated with Gram-negative bacteria, it is possible that bacterial subsets may be reduced or missing in the DSS-treated TLR4-SNP mice such that β-HB is produced to a lesser extent, thereby reducing M2a Mφ differentiation.

In conclusion, TLR4-SNP mice develop more severe DSS-colitis symptoms compared to WT mice and exhibit a reduced capacity to repair colonic damage. IL-4Rα and myeloid PPARγ are necessary for induction of M2a Mφ and we show herein that IL-4Rα and PPARγ (on myeloid cells) are necessary to repair DSS-induced colonic damage. DSS-treated TLR4-SNP mice exhibited reduced M2a Mφ markers including PPARγ but responded to rosiglitazone, providing a potential avenue for future therapeutic development. In the context of personalized medicine, we anticipate that genotyping of IBD patients for the inheritance of TLR4 SNPs may ultimately lead to the identification of a patient subset for which M2a-inducing therapies are likely to be most effective.

## MATERIALS AND METHODS

### Mice

Six-week-old WT C57BL/6J and BALB/cByJ mice were purchased from the Jackson Laboratory (Bar Harbor, ME) and acclimated in our vivarium until use at 8–10 weeks of age. TLR4-SNP mice were engineered on a C57BL/6J background to express *Tlr4* SNPs encoding D298G and N397I in murine TLR4 that are homologous to human TLR4 SNPs that encode D299G and T399I (14). IL-4Rα^−/−^ mice (BALB/c background) were originally provided by Nancy Noben-Trauth (Rockville, MD) and William Paul (NIH, Bethesda, MD). PPARγ^cKO^ mice (C57BL/6 background) were provided by Dr. Mary Jane Thomassen (East Carolina University). Originally, homozygous floxed PPARγ mice were crossed into a transgenic mouse strain on a C57BL/6 background containing the *Cre* gene under control of the Lysozyme M (*Lyz2*) promoter (PPARγ flox^+/+^/Cre^+/+^) to delete the *Pparg* gene in lysozyme-producing cells (e.g., Mφ, neutrophils) (133, 147 – 149). All transgenic strains were bred homozygously at the University of Maryland, Baltimore (UMB). Animal experiments were conducted with institutional IACUC approvals from UMB. TLR4-SNP mice are available upon request.

### DSS-induced colitis; measurements of colonic and histologic scores

Control mice received standard hyperchlorinated drinking water from Days 0 to 14. Experimental mice received 3% DSS (CAS 9011-18-1, MP Biomedicals 160110) in drinking water on Days 0–7 (changed on alternate days) (75, 77). Each new lot of DSS was titrated to ensure equivalent potency. On Day 8, all mice were provided regular drinking water only to allow a period of recovery through Day 14. A “colonic symptom score” was measured on alternate days by combining the stool consistency score (0 = normal; 1 = soft; 2 = very soft; 3 = diarrhea) and the fecal occult blood score [0 = no blood (−occult); 2 = microscopic blood in stool (+occult); 3 = macroscopic visible blood in stool (+occult); 4 = gross rectal bleeding] (75, 77). Fecal occult blood was measured using the Fisher Healthcare Sure-Vue Fecal Occult Blood Test (Fisher Scientific). At the indicated times, colons were resected, cleared of feces, and measured in centimeters distally from the cecum to just prior to the rectum. For most experiments, the most distal 1 cm colon section from control and experimental mice was opened longitudinally and fixed in 4% paraformaldehyde for 2 h at room temperature and sent to the University of Maryland School of Medicine’s Pathology Histology Core or to the University of Maryland School of Medicine’s and Greenebaum Comprehensive Cancer Center’s Pathology Biorepository Shared Services (housed in the Center for Innovative Biomedical Research (CIBR)) for sectioning and/or H&E-staining. At least two sections per mouse and >3 areas per section were analyzed using the ECHO Revolve microscope under 10× magnification for histology scoring or 100× magnification for high-power cellular counts. The total histology score represents the sum of epithelial damage (0–3), mucosal inflammatory infiltrate (0–3), submucosal inflammatory infiltrate (0–3), and muscularis externa inflammatory infiltrate (0–3), each multiplied by the respective extent of damage (1–3) (77). Under high power, the number of monocytic cells and the number of granulocytic cells were recorded in >6 high-power fields per mouse. In some experiments, the DC was flash frozen and subsequently homogenized in 0.5 or 1-mL PBS for Western analysis. The next most distal 1 cm of the colon (medial colon, MC) was flash frozen and used for RNA extraction for qRT-PCR.

### Measurement of colonic permeability

On Days 6 or 13, control or DSS-treated mice were fasted overnight before administration of 0.6 mg/g FITC-dextran in PBS (CAS 60842-46-8, Sigma-Aldrich) by oral gavage on Days 7 and 14. Mice were bled 4 h after FITC-dextran administration. Fluorescence was measured in the serum of each mouse (diluted 1:2 in 1× PBS) and compared to a standard curve (0–8,000 ng/mL FITC-dextran) in a black well plate using a fluorescent spectrophotometer (Thermo Electron Corporation Fluoroskan Ascent FL) with 485 nm excitation and 527 nm emission (186).

### Gene expression analysis

Flash-frozen colonic tissue taken from the MC was homogenized in TriPure (Roche) and RNA extracted using the manufacturer’s protocol. Since DSS has been reported to interfere with the reverse transcriptase reaction (187), RNA was purified using the LiCl purification method (188). cDNA was synthesized by reverse transcription reaction from 1 µg RNA per sample using the qScript cDNA synthesis kit (Quantabio). The 7500 Fast Real-Time PCR System and software (Applied Biosystems) was used to perform qRT-PCR (189). Forward and reverse primers included those corresponding to the housekeeping gene, *Hprt*, and M2a markers, *Chil3*, *Arg1*, *Mrc1*, and *Pparg* (Sigma) (Table S1). Primers were designed to cross intron-exon boundaries, and sequences were confirmed to target the gene of interest by NCBI BLAST. Sample amplification was verified using a negative control containing no cDNA. mRNA levels were normalized to relative *Hprt* mRNA levels and were reported using the −Δ*Ct* method (14, 190).

### Measurement of β-HB

Serum from each mouse was collected at the indicated times and filtered using 10 kDa centrifugal filter units (Millipore). β-HB was measured using the β-HB (ketone body) colorimetric assay kit (Cayman Chemical; 700190). β-HB standards were prepared according to the manufacturer’s protocol. Standards and samples (50 µL) were added to the plate in duplicate. Developer solution (50 µL) was added to each well, and the plate was incubated in the dark for 30 min at 25°C. Absorbance was measured at 450 nm using a ELx808 (BioTek) plate reader.

### Western analysis

The DC was homogenized in 0.5 or 1 mL 1× PBS and centrifuged at 1,000 × *g* for 10 min at 4°C to remove cellular debris. The supernatants were aliquoted and frozen at −20°C for Western analysis (191). Primary anti-PPARγ rabbit polyclonal antibody (Abclonal A0270; 1:2,000 dilution), primary mouse monoclonal anti-Arginase 1 (BD Biosciences 610709; 1:1,000 dilution), primary rabbit polyclonal anti-Ym1 (StemCell 60130; 1:1,000 dilution), primary goat polyclonal anti-Mrc1 (R&D Systems AF2535-SP; 1:1,000 dilution), primary rabbit anti-GAPDH (14C10) monoclonal antibody (Cell Signaling 2118; 1:1,000 dilution), secondary peroxidase-conjugated goat anti-rabbit IgG antibody (Jackson ImmunoResearch 111-035-003; 1:5,000 dilution), secondary peroxidase-conjugated goat anti-mouse IgG light chain-specific antibody (Jackson ImmunoResearch 115-035-174; 1:5,000 dilution), and secondary peroxidase-conjugated rabbit anti-goat IgG antibody (Jackson ImmunoResearch 305-035-003; 1:5,000 dilution) were used to detect species-specific primary antibodies. Proteins were detected using Pierce ECL (or ECLPlus) Western Blotting Substrate (Thermo Scientific). Densitometry was performed on scanned images of the developed film using the open-source ImageJ software (NIH). Pixel intensity across the length of each lane was calculated to create a graph of intensity vs position. The software was used to calculate the area under these curves to provide a measure of total protein present in an individual band. These calculated protein measurements were normalized to the areas under the curves for the corresponding housekeeping protein GAPDH.

### Isolation and cultivation of peritoneal Mφ

Thioglycollate-elicited Mφ (192) were treated for 24 h with medium or IL-4 (5 ng/mL) (184). Supernatants from lysates of the cells were analyzed by Western blot analysis.

### Statistical analysis

Mean values ± standard error of the mean are shown. Comparisons among more than two groups were analyzed using a two-way ANOVA with Sidak’s multiple comparisons test (GraphPad Prism 9) unless otherwise noted. Comparisons among exactly two groups were analyzed using an unpaired two-tailed Student’s *t-*test (GraphPad Prism 9). *P < 0.05; **P < 0.01; ***P < 0.001; ****P < 0.0001. The number and sex of mice per treatment are indicated in the figure legends, and individual data points are presented from the indicated number of experiments. Biostatistical services were provided by CIBR (UMB).

## Data Availability

All primary data are available from the corresponding author on reasonable request.

## SUPPLEMENTAL MATERIAL

Table S1 mbio.01208-23-s0001.docx Forward and reverse qRT-PCR primer sequences.

ASM does not own the copyrights to Supplemental Material that may be linked to, or accessed through, an article. The authors have granted ASM a non-exclusive, world-wide license to publish the Supplemental Material files. Please contact the corresponding author directly for reuse.
