# Supplementary material for: M2a macrophages facilitate resolution of chemically-induced colitis in TLR4-SNP mice
Source: mBio. 2023 Sep 28;14(5):e01208-23. doi: 10.1128/mbio.01208-23 (PMC10653841; doi:10.1128/mbio.01208-23)
Supplement: Table S1 — Forward and reverse qRT-PCR primer sequences. [file mbio.01208-23-s0001.docx]

**Table S1. Forward and reverse qRT-PCR primer sequences.**

| **Target Gene in Mouse** |  | **Sequence** |
| --- | --- | --- |
| HPRT (*Hprt*) | Forward | 5’-GCTGACCTGCTGGATTACATTAA-3’ |
|  | Reverse | 5’-TGATCATTACAGTAGCTCTTCAGTCTGA-3’ |
| Arginase1 (*Arg1*) | Forward | 5’-CAGAAGAATGGAAGAGTCAG-3’ |
|  | Reverse | 5’-CAGATATGCAGGCAGGGAGTCACC-3’ |
| Ym1 (*Chil3*) | Forward | 5’-TCTGGGTACAAGATCCCTGAA-3’ |
|  | Reverse | 5’-TTTCTCCAGTGTAGCCATCCTT-3’ |
| Mannose receptor (*Mrc1*) | Forward | 5’-GATATGAAGCCATGTACTCCTTACTGG-3’ |
|  | Reverse | 5’-GGCAGAGGTGCAGTCTGCAT-3’ |
| IL-4Rα (*Il4ra*) | Forward | 5’-TCTGCATCCCGTTGTTTTGC-3’ |
|  | Reverse | 5’-GCACCTGTGCATCCTGAATG-3’ |
